# Editorial: tRNA and protein synthesis in microorganisms

**DOI:** 10.3389/fmicb.2024.1509450

**Published:** 2024-10-30

**Authors:** Matthieu G. Gagnon, Jiqiang Ling

**Affiliations:** ^1^Department of Microbiology and Immunology, University of Texas Medical Branch, Galveston, TX, United States; ^2^Department of Biochemistry and Molecular Biology, University of Texas Medical Branch, Galveston, TX, United States; ^3^Sealy Center for Structural Biology and Molecular Biophysics, University of Texas Medical Branch, Galveston, TX, United States; ^4^Institute for Human Infections and Immunity, University of Texas Medical Branch, Galveston, TX, United States; ^5^Department of Cell Biology and Molecular Genetics, The University of Maryland, College Park, MD, United States

**Keywords:** tRNA, ribosome, tRNA modifications, genetic code, antimicrobial

## Introduction

Protein synthesis is a central process in microorganisms and is heavily targeted by antimicrobials. Studies of transfer RNAs (tRNAs), aminoacyl-tRNA synthetases (aaRSs), and ribosomes have led to deciphering the genetic code and have boosted diverse research fields. Today, research at the frontiers of tRNA biology and protein synthesis continues to provide fundamental knowledge and powerful tools for understanding the molecular basis of gene expression, microbial stress responses, microbial pathogenesis, drug design, and synthetic biology. The current Research Topic brings together some of latest advances in studies of tRNAs, aaRSs, and ribosomes in microorganisms.

## Aminoacyl-tRNA synthesis

AaRSs attach amino acids to the corresponding tRNAs, and the resulting aminoacyl-tRNAs (aa-tRNAs) are used by the ribosome to make proteins (Ibba and Söll, 2000). Many aaRSs also use editing to prevent mistranslation (Ling et al., 2009). Three articles describe the contributions of aminoacylation and editing defects to antibiotic persistence (Wood et al.), how tRNA identity elements affect aaRS editing (Cruz and Vargas-Rodriguez), and the impact of multi-aaRS complex formation on editing (Watkins et al.).

Wood et al. investigated the functional effects of methionyl-tRNA synthetase (MetRS) mutations that increased antibiotic persistence. Elegant cellular and biochemical experiments show that these MetRS mutations decrease translation initiation rates and impair editing against a key metabolite homocysteine. This work highlights that aminoacylation and editing defects could contribute to antibiotic persistence individually or in combination.

Cruz and Vargas-Rodriguez reviewed progress on how tRNAs are recognized during editing. Most aaRSs use the same tRNA identity elements during aminoacylation and editing with a few exceptions. To achieve tRNA specificity, some *trans-*editing domains (e.g., YbaK) form a complex with tRNA-binding proteins, whereas others have evolved to recognize the nucleotides near the amino acid moiety (e.g., ProXp-ala and DTD).

Watkins et al. investigated how protozoans maintain aminoacylation fidelity, which has been understudied. The life cycle of *Trypanosoma brucei* (*Tb*) poses a challenge for this protozoan to prevent the accumulation of misacylated Ala-tRNA^Pro^. Using rigorous biochemical analyses, the authors demonstrate that both prolyl-tRNA synthetase and a protein associated with the multi-aminoacyl-tRNA synthetase complex (MSC3) hydrolyze Ala-tRNA^Pro^ in *Tb*. Such an idiosyncratic proofreading machinery in protozoans may be explored as a novel drug target.

## tRNA modifications

Modifications play critical roles in regulating tRNA stability, translational efficiency and fidelity, and cellular responses (Suzuki, 2021). Three articles describe how tRNA modifications shape the evolution of codon usage in proteobacteria (Delgado et al.), the tRNA modification landscape in intracellular pathogens (Quaiyum et al.), and a comprehensive pattern of tRNA modifications at positions 34 and 37 (Masuda and Hou).

Delgado et al. performed comparative genomic analyses of codon usage in proteobacteria and uncovered a surprising link between tRNA modifications and codon evolution. Initial analysis revealed that some codons displayed a narrow range of usage frequency, and further work showed a strong correlation between changes in codon usages and the presence of tRNA modification genes, but not the number of tRNA genes. This study provides an interesting hypothesis regarding the evolution of codon usage.

Our knowledge of species-specific tRNA modification genes is limited. Quaiyum et al. predicted over 20 tRNA modification genes in Gram-negative pathogens. It appears that both the number of tRNA modifications and modification genes are reduced in *Bartonella* compared to *Escherichia coli*, suggesting that tRNA modifications have evolved to adapt to specific environments. This work also provides a paradigm pipeline to identify tRNA modification genes in other organisms.

Post-transcriptional modifications at the wobble position 34 can either expand or restrict the decoding capacity of tRNAs, while modifications at position 37 usually stabilize stacking with the preceding nucleotide. Masuda and Hou integrate the entire set of tRNA modifications in *E. coli* at positions 34 and 37 into the table of the genetic code, providing an easily accessible one-stop resource to users. The level of modification is altered in response to stress (Chionh et al., 2016; Jaroensuk et al., 2016), underlining the notion that tRNA modifications play a role in bacterial adaptation and pathogenicity.

## Ribosomes

Multilayer regulation of translation ensures that microorganisms survive under diverse environmental conditions. Three studies describe mechanisms of ribosome rescue (Teran et al.), discuss hibernation of biological molecules, including ribosomes (Helena-Bueno et al.), and raise the compelling possibility of using antibiotics to target not only translating but also hibernating ribosomes (Ekemezie and Melnikov).

During protein synthesis, ribosomes stall on mRNAs (Kurita and Himeno, 2022). Stalled ribosomes are recognized by rescue systems, including the universal transfer-messenger RNA (tmRNA) apparatus that frees ribosomes through trans-translation. Teran et al. reported the cryo-EM structure of a ribosome rescue complex isolated from an *E. coli* lysate and containing endogenous tmRNA, the small protein B (SmpB), and tRNA^Ala^ in the ribosomal A site. The structure, which represents the first decoding event of the alanine codon (GCA) within the messenger-like domain (MLD) of tmRNA, reveals that the accommodation of tRNA^Ala^ rearranges the adenosine-rich linker of tmRNA. This flexible linker, previously observed to partially block the incoming tRNA (Rae et al., 2019; Guyomar et al., 2021), interacts with the anticodon stem of tRNA^Ala^.

Under conditions of starvation and stress, organisms protect their biological molecules through hibernation. Helena-Bueno et al. reviewed the concept of hibernation of biological molecules. Remarkably, in addition to hibernation factors, “canonical” translation factors also participate in ribosome hibernation, such as eEF2 and eIF5A in eukaryotes, and EF-Tu in bacteria. Other cellular enzymes, like RNA polymerase in both yeast and bacteria bind to Rrn3 in eukaryotes or HelD in bacteria and inactivate the enzyme during starvation and stress. During darkness, Rubisco in plants binds to the small molecule inhibitor 2-carboxy-D-arabinitol 1-phosphate (CA1P), turning off photosynthesis.

The ribosome is a major antibiotic target. Ekemezie and Melnikov reviewed the potential role of ribosome hibernation factors in antibiotic resistance. The observation that several ribosome-associated hibernation factors overlap with drug-binding sites led to the question of how ribosome hibernation influences antibiotic efficacy. The possibility that hibernating molecules enable pathogenic bacteria to withstand assaults from antibiotics could open possibilities to effectively combat infections by disrupting pathogens' mechanisms of molecular hibernation by targeting sleeping enzymes.
